# DNMT3A and DNMT3B Targeting as an Effective Radiosensitizing Strategy in Embryonal Rhabdomyosarcoma

**DOI:** 10.3390/cells10112956

**Published:** 2021-10-30

**Authors:** Simona Camero, Giulia Vitali, Paola Pontecorvi, Simona Ceccarelli, Eleni Anastasiadou, Francesca Cicchetti, Elisabetta Flex, Silvia Pomella, Matteo Cassandri, Rossella Rota, Francesco Marampon, Cinzia Marchese, Amalia Schiavetti, Francesca Megiorni

**Affiliations:** 1 Department of Maternal, Infantile and Urological Sciences, Sapienza University of Rome, Viale Regina Elena 324, 00161 Rome, Italy; simona.camero@uniroma1.it (S.C.); amalia.schiavetti@uniroma1.it (A.S.); 2 Department of Experimental Medicine, Sapienza University of Rome, Viale Regina Elena 324, 00161 Rome, Italy; vitali.1768362@studenti.uniroma1.it (G.V.); paola.pontecorvi@uniroma1.it (P.P.); simona.ceccarelli@uniroma1.it (S.C.); eleni.anastasiadou@uniroma1.it (E.A.); cinzia.marchese@uniroma1.it (C.M.); 3 Policlinico Umberto I Hospital, Viale del Policlinico 155, 00161 Rome, Italy; F.Cicchetti@policlinicoumberto1.it; 4 Department of Oncology and Molecular Medicine, Istituto Superiore di Sanità, Viale Regina Elena 299, 00161 Rome, Italy; elisabetta.flex@iss.it; 5 Group of Epigenetics of Pediatric Sarcomas, Department of Oncohematology, Bambino Gesù Children’s Hospital, IRCCS, Via San Paolo 15, 00146 Rome, Italy; silvia.pomella@opbg.net (S.P.); matteo.cassandri@opbg.net (M.C.); rossella.rota@opbg.net (R.R.); 6 Department of Radiological, Oncological and Pathological Sciences, Sapienza University of Rome, Viale Regina Elena 324, 00161 Rome, Italy

**Keywords:** rhabdomyosarcoma, DNMT3A, DNMT3B, radiotherapy, target therapies, RNA interference, DNA damage, differentiation therapies, senescence

## Abstract

Rhabdomyosarcoma (RMS) is the most common soft tissue sarcoma in childhood. Recently, we demonstrated the overexpression of both DNA methyltransferase 3A (DNMT3A) and 3B (DNMT3B) in RMS tumour biopsies and cell lines compared to normal skeletal muscle. Radiotherapy may often fail due to the abnormal expression of some molecules able to drive resistance mechanisms. The aim of this study was to analyse the involvement of DNMT3A and DNMT3B in radioresistance in RMS. RNA interference experiments against DNMT3A/3B were performed in embryonal RMS cells, upon ionizing radiation (IR) exposure and the effects of the combined treatment on RMS cells were analysed. DNMT3A and DNMT3B knocking down increased the sensitivity of RMS cells to IR, as indicated by the drastic decrease of colony formation ability. Interestingly, DNMT3A/3B act in two different ways: DNMT3A silencing triggers the cellular senescence program by up-regulating p16 and p21, whilst DNMT3B depletion induces significant DNA damage and impairs the DNA repair machinery (ATM, DNA-PKcs and Rad51 reduction). Our findings demonstrate for the first time that DNMT3A and DNMT3B overexpression may contribute to radiotherapy failure, and their inhibition might be a promising radiosensitizing strategy, mainly in the treatment of patients with metastatic or recurrent RMS tumours.

## 1. Introduction

Rhabdomyosarcoma (RMS) represents about 3–5% of malignant solid tumours in the paediatric population and is the most common soft tissue sarcoma in children. RMS originates from myogenic precursors, which have lost the control in cell growth and differentiation [1] and is characterized by a high grade of malignancy, local invasiveness, and a marked propensity to metastasize [2]. The two main histological subtypes, alveolar RMS (ARMS) and embryonal RMS (ERMS), have specific molecular alterations and outcomes. ARMS tumours are more frequent in children and adolescents and are characterized by a more aggressive course, with metastases already diffuse at diagnosis and a poor prognosis, whilst ERMSs arise especially in infants and young children and are generally correlated with a better outcome [1,3]. The current treatments for early and local RMS, including surgery, chemotherapy, and radiotherapy, frequently fail, resulting in disease progression and relapse [4,5]. Radiation therapy, based on ionizing radiations (IR), promotes therapeutic effects against tumour cells inducing apoptosis, necrosis, mitotic catastrophe, senescence, and autophagy by directly triggering DNA double-strand breaks (DSBs) or by indirectly stimulating the formation of reactive oxygen species (ROS) [6]. However, cancer cells, through several mechanisms, including DNA damage response (DDR) and antioxidant activity, may escape from cell death programs induced by radiotherapy [7,8]. Therefore, understanding all the factors and the molecular pathways involved in the radioresistance mechanisms is crucial for the development of innovative therapies, more effective and less toxic for patients with RMS tumours.

Epigenetic alterations have been demonstrated to play a key role in onset and progression of several human cancers, including RMS, especially through the transcriptional repression of tumour suppressor genes [9,10,11,12,13,14,15]. Since epigenetic mechanisms control chromatin structure and gene expression [16], which are both associated with the response to IR, several studies have recently investigated the inhibition of histone deacetylases (HDACs) and DNA methyltransferases (DNMTs) as potential radiosensitizing approaches against cancer growth and spreading [17,18,19,20]. Indeed, treatment with Belinostat, a new pan-HDAC inhibitor, is able to increment the efficacy of radiotherapy in ARMS and ERMS cell lines and in xenograft mouse models [21]. Moreover, knocking down of DNMT3B expression has been demonstrated to sensitize prostate cancer, nasopharyngeal carcinoma, and oesophageal cancer to radiation [22,23,24]. Our group also reported DNMT3A and DNMT3B overexpression in RMS tumour primary biopsies and cell lines compared to normal skeletal muscle, so revealing the oncogenic role of DNMT3B in ERMS cell lines [25]. To date, no study has been conducted to assess whether DNMT3A and DNMT3B silencing could increase the sensitivity to IR in RMS. Here, we demonstrate that the preliminary knocking down of DNMT3A or DNMT3B genes significantly promotes radiosensitivity in ERMS cellular models by affecting senescence- and DNA repair-related pathways, respectively.

## 2. Materials and Methods

### 2.1. Cell Cultures and Compounds

In vitro models of ERMS were used in this study. Specifically, the RD cell line was purchased from ATCC (CCL-136^TM^), whilst the JR1 cell line was kindly provided by Dr. Rota R (Bambino Gesù Children’s Hospital, Rome, Italy). All the cell lines were maintained in complete medium, i.e., high-glucose DMEM (DMEM-HG, Sigma-Aldrich, St. Louis, MO, USA), supplemented with 10% foetal bovine serum (FBS) (Corning, New York, NY, USA), 2 mM l-glutamine (Corning), 100 IU/mL penicillin and 100 μg/mL streptomycin (Corning) and grown at 37 °C in 5% CO_2_.

SB203580, a p38-specific inhibitor, was purchased from Selleckchem (Suffolk, UK) as lyophilized powders and were reconstituted in DMSO to a final concentration of 5 mM. SB203580 was added to the cultured medium 24 h after specific transfection to a final concentration of 5 μM.

### 2.2. Transient Transfection of Small Interfering RNA and Radiation Exposure

ERMS cells were seeded at 1.5 × 10^5^ cells/well in 12-well plates; small interfering RNA (siRNA) against human DNMT3A, DNMT3B or siRNA negative control (si-DNMT3A, sc-37757; si-DNMT3B, sc-37759; si-NC, sc-37007 by Santa Cruz Biotechnology, Dallas, TX, USA) were combined with RNAiMAX (Invitrogen, Carlsbad, CA, USA) at 60 nM final concentration following the manufacturer’s protocol. si-DNMT3A and si-DNMT3B are a pool of 3 target-specific 19–25 nt siRNAs designed to specifically knock down gene expression. Forty-eight h after transfection, ERMS cells were irradiated using an ONCOR Impression Linear Accelerator (Siemens Medical Solutions USA, Inc, Concord, CA, USA) at a single dose of 4 Gy (380 UM/min). Cells were collected at different time points after transfection/irradiation for the subsequent assays.

### 2.3. Cell Morphological Evaluation by Giemsa Staining

To observe the morphological changes of ERMS cells irradiated and transfected with si-DNMT3A, si-DNMT3B, or si-NC, cells were stained by the standard Giemsa method 144 h post-transfection and 96 h after radiation exposure. Briefly, cells were fixed by cold methanol for 15 min and stained in 10% Giemsa solution (Sigma-Aldrich, St. Louis, MO, USA) for 15 min at RT. Cells in four random fields for treatment were photographed at 20× magnification with EVOS XL Core Imaging System (Thermo Fisher Scientific, Waltham, MA, USA). Experiments were performed two time.

### 2.4. Cell Proliferation and MTT Assays

RD cells at 96 h after radiation exposure (4 Gy) and 144 h after siRNA transfection (si-DNMT3A, si-DNMT3B or si-NC) were labelled with trypan blue (Invitrogen) in a 1:1 mixture and counted using the Countess II Automated Cell Counter (Invitrogen), according to the manufacturer’s instructions.

For MTT assays, 24 h before the transient transfection with the specific siRNA against DNMTs or the negative control, RD cells were plated at 1.0 × 10^4^ cells/well in 96-well plates and after additional 48 h were exposed or not to radiation. At different time points after irradiation (0–48–72 h), the cell viability was analysed by using MTT [3-(4,5-dimethylthiazol 2-yl)-2,5-diphenyltetrazolium] assay. Specifically, 10 μL of MTT (5 mg/mL, Sigma-Aldrich) were added to each well and plates were incubated at 37 °C for 3 h. Media were then replaced with 100 μL of DMSO and RD cells were incubated at 37 °C for 10 min. Blank cell-free wells were also included. Absorbance was read at wavelength of 560 nm by using GloMax Discover Microplate reader (Promega, Madison, WI, USA). The results were plotted as means ± SD of two independent experiments each performed in triplicate.

### 2.5. Trans-Well Migration Assay

For the cell migration assay, RD cells at 3 h after radiation exposure were plated in serum-free DMEM-HG at 7.5 × 10^4^ cell/well in the upper compartment of the BD Falcon^TM^ Cell Culture Inserts with 8 μm pore polycarbonate filters (Falcon, New York, NY, USA) placed into a 24-well culture plate. The lower compartment contained DMEM-HG with 10% FBS, used as chemoattractant. After overnight (ON) incubation at 37 °C migrated cells were fixed in 100% methanol and stained with 1% crystal violet dye. Non-migrating cells on the upper surface of the membrane were removed with cotton swabs. Five random fields were photographed with EVOS XL Core Imaging System (Thermo Fisher Scientific) at 10× magnification and the number of cells was calculated by using ImageJ software [26]. Data were plotted as mean ± SD of migrated cells of three independent experiments each performed in duplicate.

### 2.6. RNA Extraction and Quantitative Real Time PCR (q-PCR)

Total RNA was isolated from RD cells at 3, 24, or 96 h after radiation exposure using TRIsure^TM^ (BIOLINE, London, UK) according to the manufacturer’s instructions. cDNA was obtained and analysed as previously described [15]. Transcript levels of DNMT3A, DNMT3B, and MYHC genes were quantified using specific TaqMan Real-Time Gene Expression Assays (Applied Biosystems, Foster City, CA, USA). The mRNA expression levels of CDKN1A, MYOD and MYOGENIN were analysed with the SensiFAST SYBR Hi-ROX Kit (BIOLINE). All samples were normalised according to GAPDH transcript levels. Expression levels of human miR-106b, miR-17-5p, miR-133a, and miR-206 were quantified using TaqMan MicroRNA Reverse Transcription Kit (Applied Biosystems) and miRNA-specific TaqMan MGB primers/probe (Life Technologies, Carlsbad, CA, USA). U6 small nuclear RNA levels were used as an internal control. The relative amount of each mRNA or miRNA was calculated by the comparative Ct method [27] and expressed as fold change in comparison to negative control cells. Each sample was run in triplicate, in at least three independent experiments.

### 2.7. Protein Extracts and Western Blot Analysis

Total proteins were extracted from ERMS cells collected at different times after siRNA transfection (48–72–144 h) and irradiation (3–24–96 h) by using RIPA buffer. For Western blotting experiments, 30–70 μg of proteins were separated on 7–15% SDS-PAGE and transferred onto PVDF membranes (Amersham, Chicago, IL, USA). Filters were blocked with 5% non-fat dry milk or 3% BSA in PBS-Tween for 1 h at RT and incubated over-night at +4 °C or 2 h at RT with the following primary antibodies: DNMT3A, cyclin B1, cyclin D1, p21, p38, Ku70, ATM, Rad51, DNA-PKcs, Bcl-2 and Caveolin-1 (Santa Cruz Biotechnology, Dallas, TX, USA), DNMT3B (GeneTex, Irvine, CA, USA), p16 (Bethyl, Montgomery, TX, USA), p-p38, γ-H2AX (Cell Signalling Technology, Danvers, MA, USA), Lamin B (Abcam, Cambridge, UK), MyoD, Myogenin and MyHC (Millipore, Burlington, MA, USA). Tubulin (Santa Cruz Biotechnology) was used as internal control. Images were acquired by ChemiDoc XRS+ (Bio-Rad, Hercules, CA, USA). All experiments were performed at least three times. Densitometric analysis was performed using the Image Lab 5.1 software (Bio-Rad) and graphed as histograms with statistical significance.

### 2.8. Cell Cycle Analysis

For cell cycle analysis, RD cells were counted at 72 h after siRNA transfection and 24 h post-radiation exposure. At least 1 × 10^6^ cells were fixed in 70% ice-cold ethanol overnight at +4 °C and then cell pellets were processed as previously described [25]. FACS data were analysed by using the ModFit LT 3.0 software (Verity Software House, Topsham, ME, USA). Experiments were performed four times.

### 2.9. Colony Formation Assay

For anchorage-dependent colony formation assay, RD cells were collected 24 h after irradiation and seeded in 6-well plates in triplicate at low density (2 × 10^3^ cells/well). Cells were incubated at 37 °C for 10 or 12 days (medium was replaced every three days) and were then fixed, stained, and photographed as previously described [15]. For quantitative analysis, crystal violet was solubilised in 30% acetic acid in water for 15 min at RT and absorbance was measured by using the Biochrom Libra S22 UV/VIS spectrophotometer (Biochrom, Berlin, Germany) at a wavelength of 595 nm. Acetic acid (30% in water) was used as blank control. The results were graphed as mean ± SD of four independent experiments each performed in triplicate.

### 2.10. Immunofluorescence

For immunofluorescence (IF) assay, RD cells (8 × 10^4^), seeded onto 2% gelatine coated-glass coverslips in 24-well plates at 144 h from siRNA transfection and 96 h from irradiation, were allowed to attach ON at 37 °C and were fixed as previously described [28]. For IF analysis, RD cells were incubated with the following primary antibodies: γ-H2AX (Cell Signalling Technology), MyHC (Millipore) and Lamin B (Abcam). Actin cytoskeleton was visualized using TRITC–Phalloidin (1:100 in PBS). DAPI (Sigma-Aldrich) was used for nuclear staining. The single-stained and merged images were acquired with a Zeiss ApoTome microscope (40× magnification) using the Axiovision software (Carl Zeiss, Jena, Germany). Merged Phalloidin/DAPI images from four random fields for each treatment were analysed using the ImageJ software, measuring for each cell both the area and perimeter of cytoplasm and nucleus (or nuclei, in case of multinucleated cells). The respective nucleus/cytoplasm (N/C) ratios were then calculated and graphed as means ± SD. IF experiments were performed two times.

### 2.11. Statistical Analysis

Data are presented as means ± SD. Statistical analyses were performed by using two-way ANOVA and considering * *p* < 0.05, ** *p* < 0.01 and *** *p* < 0.001. Tukey correction was applied for multiple comparisons. All the experiments were performed in triplicates and repeated at least three times unless mentioned otherwise.

## 3. Results

### 3.1. Radiotherapy Alone Does Not Decrease DNMT3A and DNMT3B Expression Levels

**Figure 1:**
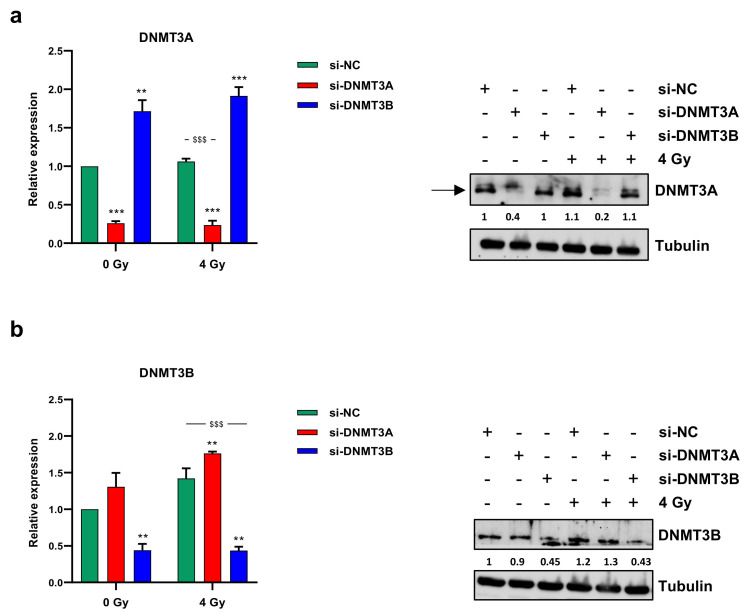
DNMT3A and DNMT3B expression levels. Transcript levels (left panels) and protein expression (right panels) of DNMT3A (**a**) and DNMT3B (**b**) in RD cells transfected with si-DNMT3A, si-DNMT3B or si-NC 48 h before being exposed or not to radiation (4 Gy). q-PCR analysis was expressed as fold increase over si-NC/0 Gy, arbitrarily set at 1, and GAPDH was used as endogenous control. Bars represent mean values ± SD of three independent experiments, each performed in triplicate. Statistical analyses were performed by using two-way ANOVA: ***, *p* < 0.001; **, *p* < 0.01 vs. si-NC/0 Gy; $$$, *p* < 0.001 vs. si-NC/4 Gy. For Western blotting assay, tubulin was used as loading control. In Figure S1a are reported the densitometric analyses of the different experiments performed and the corresponding statistical analysis.

Since we recently demonstrated the up-regulation of both DNMT3A and DNMT3B transcript levels in RMS tumour samples compared to normal skeletal muscle and the antitumoral effects of DNMT3B knocking down on ERMS cell lines [25], here, we analysed DNMT3A/3B involvement in radioresistance mechanisms. To evaluate the effects of the combined treatment of the specific DNMT silencing with irradiation, we performed RNA interference experiments by using specific small interfering RNA against DNMT3A or DNMT3B mRNA in RD cells, which were irradiated with a single dose of 4 Gy 48 h later. DNMT-specific knocking down was confirmed by q-PCR and Western blotting experiments at 72 h after transfection and 24 h after radiation exposure. As shown in Figure 1a,b, a significant reduction of DNMT3A or DNMT3B at both mRNA and protein levels was observed in si-DNMT3A and si-DNMT3B treated cells, respectively, with or without irradiation, compared to the mocked controls.

### 3.2. DNMT3A and DNMT3B Knocking down Enhance Radiotherapy-Induced Cell Proliferation Inhibition

**Figure 2:**
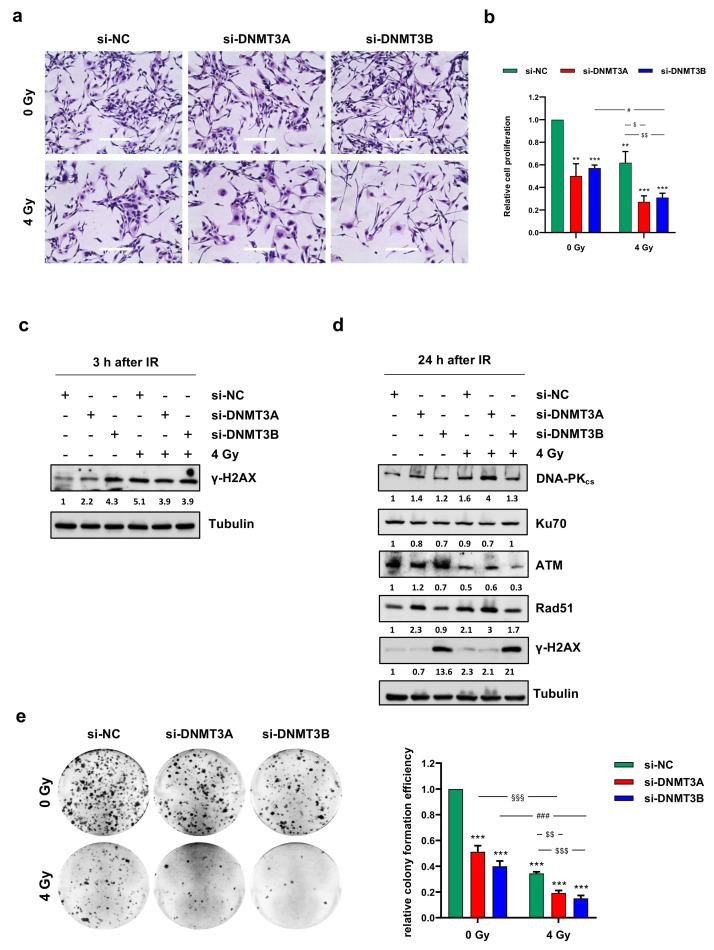
DNMT3A and DNMT3B knocking down increase sensitivity of RMS cells to radiation. (**a**) Representative images of RD cells stained with Giemsa and photographed at 20× magnification at 144 h after DNMT3A or DNMT3B depletion and 96 h after irradiation. si-DNMT3A samples showed flattened, enlarged, and irregular morphology, whilst si-DNMT3B cells displayed more elongated cellular bodies. Scale bar 200 μM. (**b**) RD cell proliferation assessed by trypan blue exclusion staining at 144 h after silencing with specific siRNA and irradiated or not with a single dose of 4 Gy. Results represent mean values ± SD of four independent experiments expressed as fold increase over negative control (si-NC/0 Gy), arbitrarily set at 1. Statistical analyses were performed by using two-way ANOVA: ***, *p* < 0.001; **, *p* < 0.01 vs. si-NC/0 Gy; #, *p* < 0.05 vs. si-DNMT3B/0 Gy; $$, *p* < 0.01; $, *p* < 0.05 vs. si-NC/4 Gy. (**c**) Western blotting showed γ-H2AX expression levels in RD cells transfected with si-DNMT3A, si-DNMT3B or si-NC at 3 h after radiation treatment. Tubulin expression was used as loading control. (**d**) Western blotting assay of specific proteins involved in DNA damage signaling and response at 24 h after irradiation. Tubulin was used as internal control. (**e**) RD cells after the combined treatment were seeded at low density and allowed to grow for 12 days. Representative pictures of colonies stained with crystal violet. Histograms represent means ± SD of five independent experiments, each performed in triplicate. Colony-forming efficiency, calculated by crystal violet absorbance, was expressed as fold increase over si-NC/0 Gy, arbitrarily set at 1. Statistical analyses were performed by using two-way ANOVA: ***, *p* < 0.001, vs. si-NC/0 Gy; §§§, *p* < 0.001 vs. si-DNMT3A/0 Gy; ###, *p* < 0.001 vs. si-DNMT3B/0 Gy; $$$, *p* < 0.001; $$, *p* < 0.01 vs. si-NC/4 Gy. Figure S1b,c reports the densitometric analyses of the different experiments performed and the corresponding statistical analysis.

To assess the ability of DNMT3A and DNMT3B silencing to synergize with radiation therapy, we analysed the effects of the combined treatment on cell proliferation. Direct counting of living cells with trypan blue dye exclusion test and Giemsa staining performed on RD cells 72 h after irradiation showed a drastic decrease of cell proliferation in DNMT3A/3B depleted RD cells exposed to ionizing radiation (IR) compared to both mocked controls and irradiated cells (Figure 2a,b).

Notably, in RD cells treated with a single dose of 4 Gy, the growth inhibition was about 40% and reached 70% with both DNMT3A and DNMT3B silencing (Figure 2b). Moreover, Giemsa staining highlighted a different morphology in si-DNMT3A cells, which showed a flattened, enlarged, and irregular morphology, whilst si-DNMT3B cells displayed more elongated cellular bodies compared to the negative control (si-NC) cells (Figure 2a). Similar results were obtained in JR1 cells, as reported in Figure S2a.

To evaluate whether the reduction of cell proliferation was due to DNA damage accumulation, we analysed the amount of phospho-H2AX (γ-H2AX), a well-established marker for DNA double strand breaks (DSBs). As expected, Western blotting assay showed γ-H2AX up-regulation at 3 h after irradiation (Figure 2c), but surprisingly, a strong activation of H2AX histone was already present in DNMT3B depleted RD cells not exposed to IR. Given the ability of tumour cells to repair DNA DSBs, we analysed the modulation of key molecules involved in DNA damage response (DDR) to assess whether the concomitant silencing of DNMTs was able to counteract the radioresistance mechanisms. Twenty-four hours after radiation exposure, DNA-PKcs, ATM, and Rad51 expression levels were markedly decreased only in si-DNMT3B/4 Gy cells (Figure 2d); in agreement with this observation, Western blotting experiments showed a persistent activation of γ-H2AX only in this sample, suggesting that after the initial induction of DNA damage, some breaks remain unrepaired. A marked induction of γ-H2AX levels in DNMT3B silenced cells with and without irradiation was also observed in JR1 cells (Figure S2b). To better understand the long-lasting effects of DNMT3A and DNMT3B knocking down on RD cells exposed to radiation, we allowed to grow the cells at very low density for 12 days to analyse their clonogenic potential. After IR treatment, the ability to form colonies was significantly reduced in all the samples compared to the negative control cells (Figure 2e; si-DNMT3A 0.51-fold; si-DNMT3B 0.40-fold; si-NC/4 Gy, 0.34-fold; si-DNMT3A/4 Gy, 0.19-fold; si-DNMT3B/4 Gy, 0.15-fold). Moreover, si-DNMT3A/4 Gy and si-DNMT3B/4 Gy cells showed a lower ability in anchorage-dependent experiments in respect to si-NC/4 Gy and si-DNMT3A or si-DNMT3B samples, respectively, and the comparison between the combined treatment vs. silencing alone (§§§; ###, *p* < 0.001) or vs. radiotherapy alone ($$, *p* < 0.01; $$$, *p* < 0.001) remained significant (Figure 2e).

To assess whether DNMT3A/3B silencing was also able to impair cell migration of irradiated cells, trans-well migration analysis was performed 24 h after IR treatment. A significant decrease of cell number was observed on the bottom side of the Boyden membranes in DNMT3B depleted RD cells, si-NC/4 Gy and si-DNMT3B/4 Gy samples compared to mocked controls (Figure S3). The comparison between si-DNMT3B/4 Gy vs. si-DNMT3B or vs. si-NC/4 Gy was not significant, indicating that DNMT3B knocking down and radiotherapy affect cell migration but do not act synergistically on its inhibition. Altogether, these results suggest that DNMT3A and DNMT3B depletion potentiates the cytostatic effect induced by IR on RD cells in two different ways: si-DNMT3A activity is independent of DNA damage, whilst si-DNMT3B exerts its effects by blocking the DNA repair machinery.

### 3.3. Cell Growth Inhibition Is Promoted by Sustained p21 Activation

**Figure 3:**
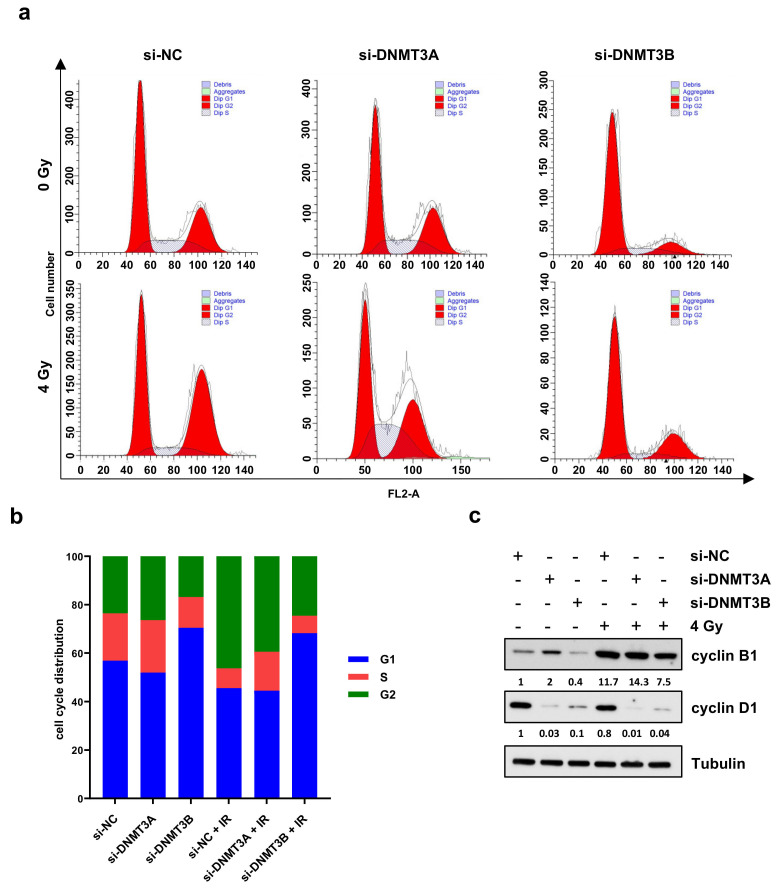
Cell cycle distribution of RD cells after DNMT3A and DNMT3B silencing and IR exposure. (**a**) Representative cell cycle distribution of RD cells at 24 h after irradiation and 72 h upon DNMT knocking down with the specific siRNAs. (**b**) Flow-cytometry assay-related bars showing cell percentage in G1, S, and G2 phases in RD cells silenced for DNMT3A or DNMT3B and exposed to radiation. Data are average value of four independent experiments. (**c**) Western blotting assays showing cyclin B1 and cyclin D1 levels in RD cells with or without specific siRNAs and IR. Tubulin was used as internal control. In Figure S1d are reported the densitometric analyses of the different experiments performed and the corresponding statistical analysis.

Based on these results, we analysed the cell cycle progression of RD cells 24 h after irradiation by using flow cytometry (Figure 3a,b).

**Figure 4:**
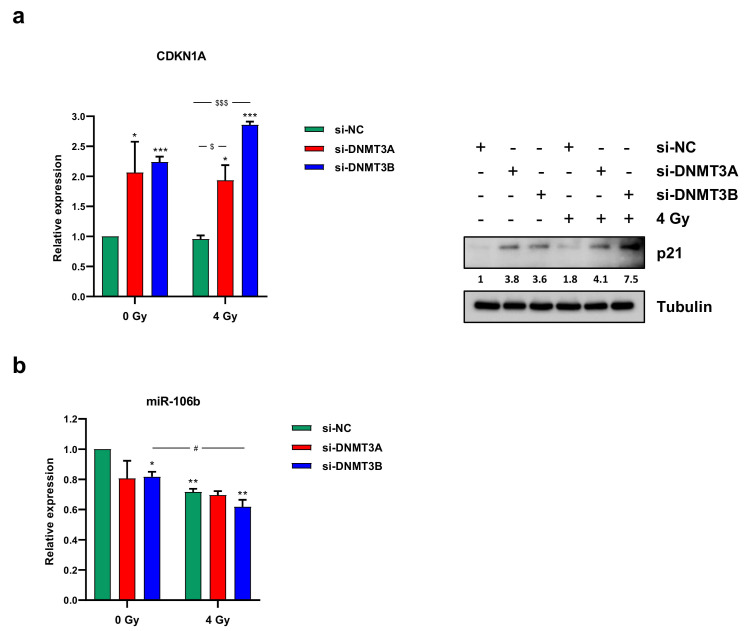
DNMT3A and DNMT3B depletion induced a marked p21 overexpression in irradiated RD cells. (**a**) mRNA (left panel) and protein levels (right panel) of p21 (CDKN1A) in RD cells after DNMT3A or DNMT3B silencing and IR exposure. q-PCR analysis was expressed as fold increase over si-NC/0 Gy, arbitrarily set at 1 and GAPDH was used as endogenous control. Bars represent means ± SD of three independent experiments, each performed in triplicate. Statistical analyses were performed by using two-way ANOVA: ***, *p* < 0.001; *, *p* < 0.05 vs. si-NC/0 Gy; $$$, *p* < 0.001, $, *p* < 0.05 vs. si-NC/4 Gy. For Western blotting assay, tubulin was used as loading control. (**b**) miR-106b expression levels analysed by q-PCR assay in RD cells after the combined treatment (silencing/irradiation) and expressed as fold increase over si-NC/0 Gy, arbitrarily set at 1. U6 was used as internal control. Histograms represent mean values ± SD of three independent experiments, each performed in triplicate. Statistical analyses were performed by using two-way ANOVA: **, *p* < 0.01; *, *p* < 0.05 vs. si-NC/0 Gy; #, *p* < 0.05 vs. si-DNMT3B/0 Gy. In Figure S1e are reported the densitometric analyses of the different experiments performed and the corresponding statistical analysis.

PI staining displayed two different patterns. DNMT3A knocking down alone did not significantly perturb the cell cycle distribution in comparison to mocked control cells (Figure 3b). The co-treatment with radiation therapy induced an increased cell percentage at G2 phase with the concomitant decrease at G1 phase in respect to si-NC and si-DNMT3A transfected RD cells (Figure 3b). After the combined si-DNMT3A and IR treatment, we also detected a marked increase of cell percentage in S phase compared to si-NC/4 Gy samples (Figure 3b). On the contrary, a drastic G1 cell cycle arrest was observed in si-DNMT3B and si-DNMT3B/4 Gy cells in comparison to both si-NC and si-NC/4 Gy (Figure 3b). These results are in accordance with our previously published data [25] and confirm the key role played by DNMT3B on G1/S transition in ERMS cells. Moreover, si-DNMT3B/4 Gy showed higher cell percentage at G2 phase than si-DNMT3B cells and a marked S phase reduction (Figure 3a,b; si-NC: G1 56.91%, S 19.53%, G2 23.56%; si-DNMT3A: G1 51.95%, S 21.64%, G2 26.41%; si-DNMT3B: G1 70.45%, S 12.71%, G2 16.84%; si-NC/4 Gy: G1 45.59%, S 8.16%, G2 46.25%; si-DNMT3A/4 Gy: G1 44.56%, S 15.97%, G2 39.47%; si-DNMT3B/4 Gy: G1 68.20%, S 7.27%, G2 24.53%). To better understand the mechanisms underlying this cell cycle perturbation, the expression of specific cell cycle markers was quantified by Western blotting. In agreement with the cytofluorimetric analysis, RD cells exposed to radiation showed a strong up-regulation of cyclin B1 (required for the G2/M transition) and a concomitant decrease of cyclin D1 (necessary for the G1/S transition) compared to the corresponding not irradiated cells (Figure 3c). In particular, si-DNMT3B/4 Gy cells exhibiting lower levels of cyclin B1 than both si-NC/4 Gy and si-DNMT3A/4 Gy samples, because they are essentially arrested at G1 phase (Figure 3b,c). Since p21 is a well-known protein able to arrest cell cycle progression at both G1/S and G2/M transition, we also investigated the effects of the DNMT3A/3B silencing and IR treatment on p21 expression. As shown by q-PCR (Figure 4a, left panel) and Western blotting assays (Figure 4a, right panel), p21 transcript and protein levels were significantly up-regulated in DNMT3A and DNMT3B silenced cells compared to the mocked controls. Interestingly, radiation therapy acted synergistically only in the presence of DNMT3B depletion, with si-DNMT3B/4Gy cells displaying the greatest increase of p21 levels. Regarding DNMT3A knocking down, no significant difference was observed in DNMT3A depleted cells with or without irradiation. Up-regulation of the p21 protein levels was also confirmed in JR1 cells exposed to IR after the specific silencing (Figure S2c).

Since we found p21 up-regulation without p53 activation after the combined treatment (data not shown), and considering that i) RMS, as many other malignancies, is characterised by mutation and inactivation of p53 tumour suppressor gene [29], and ii) the ability of DNMTs to control miRNA expression, we wondered if a p21 increase was due to the down-regulation of specific miRNA. The abundance of miR-106b and miR-17-5p, which are able to target p21, was analysed by q-PCR at 3 h after radiation exposure. Whilst we did not detect miR-17-5p deregulation (data not shown), a decrease of miR-106b levels was induced by DNMT3B silencing and IR combined treatment (Figure 4b). Altogether, these results suggest that the persistence of γ-H2AX foci induced by DNMT3B knocking down leads to a drastic G1 cell cycle arrest promoted by persistent p21 activation.

### 3.4. γ-H2AX Up-Regulation and the Reduced Clonogenic Capacity Induced by DNMT Knocking down Are Mediated by p38 Activation

**Figure 5:**
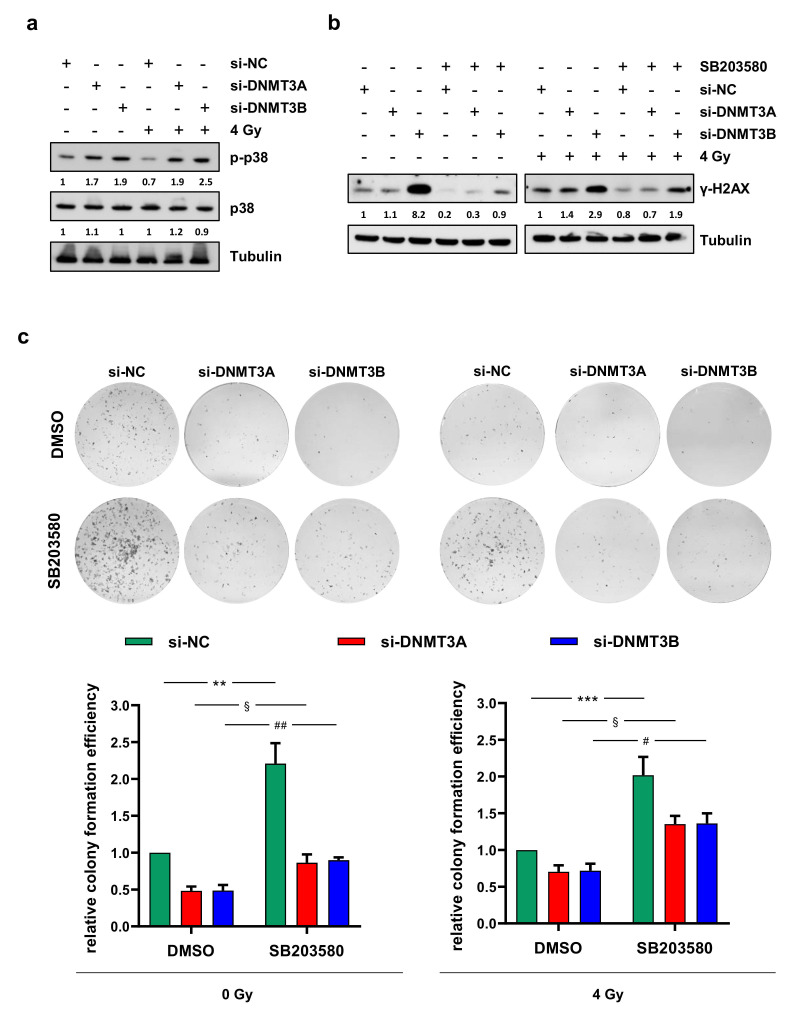
p38 inhibition counteracts γ-H2AX up-regulation and the reduced clonogenic ability induced by DNMT3A/3B silencing. (**a**) Western blotting showing the increase of phosphorylated p38 (p-p38) in RD cells at 24 h upon irradiation and 72 h after DNMT3A or DNMT3B knocking down. Levels of total p38 was not perturbed by the specific siRNA transfection and/or irradiation. Tubulin expression was used as loading control. (**b**) Western blotting showing γ-H2AX levels in RD cells pre-treated with SB203580 or DMSO, transfected with si-DNMT3A, si-DNMT3B or si-NC, and exposed or not to radiation. Tubulin expression was used as internal control. (**c**) RD cells at 24 h after irradiation were seeded at low density and allowed to grow for 10 days. Representative images of colonies stained with crystal violet. Bars represent means ± SD of three independent experiments, each performed in triplicate. Colony forming efficiency, calculated by crystal violet absorbance, was expressed as fold increase over si-NC/DMSO/0 Gy (left panel) and si-NC/DMSO/4 Gy (right panel) arbitrarily set at 1. Statistical analyses were performed by using two-way ANOVA: **, *p* < 0.01, vs. si-NC/DMSO/0 Gy; §, *p* < 0.05 vs. si-DNMT3A/DMSO/0 Gy; ##, *p* < 0.01 vs. si-DNMT3B/DMSO/0 Gy (left panel); ***, *p* < 0.001, vs. si-NC/DMSO/4 Gy; §, *p* < 0.05 vs. si-DNMT3A/DMSO/4 Gy; #, *p* < 0.05 vs. si-DNMT3B/DMSO/4 Gy (right panel). Figure S1f,g presents the densitometric analyses of the different experiments performed and the corresponding statistical analysis.

Since p38 might play a key role in response to DNA damage in p53-defective cells [30] and considering the increased expression levels induced by DNMT3B knocking down [25], we analysed the activation status of this mitogen-activated protein kinase (MAPK) after DNMT3A or DNMT3B silencing and radiation exposure. As shown in Figure 5a, increased levels of phosphorylated p38 (p-p38) were observed in both DNMT3A- and DNMT3B-depleted RD cells and exposed or not to IR for 24 h. The expression of total p38 protein was similar in the different samples (Figure 5a).

In order to understand if the up-regulation of p-p38 was responsible for DNA damage repair or its inhibition, we analysed the ability to form colonies and the abundance of γ-H2AX protein in RD cells (i) transfected with si-DNMT3A, si-DNMT3B, or si-NC (ii) treated with the p38 inhibitor SB203580 or DMSO as a negative control and (iii) exposed or not to a single dose of 4 Gy. As shown in Figure 5b, preincubation of RD cells with SB203580 determined a drastic decrease of γ-H2AX expression levels compared to DMSO mocked cells. In accordance with this result, p38 inhibition counteracted the reduction of clonogenic potential previously observed in RD cells transfected with si-DNMT3A and si-DNMT3B with and without irradiation compared to si-NC samples (Figure 5c).

These results suggest that the inhibition of p38 MAPK activity might contribute to radiotherapy resistance observed in RMS.

### 3.5. DNMT3A Depletion Stimulates Senescence in RMS Cells

**Figure 6:**
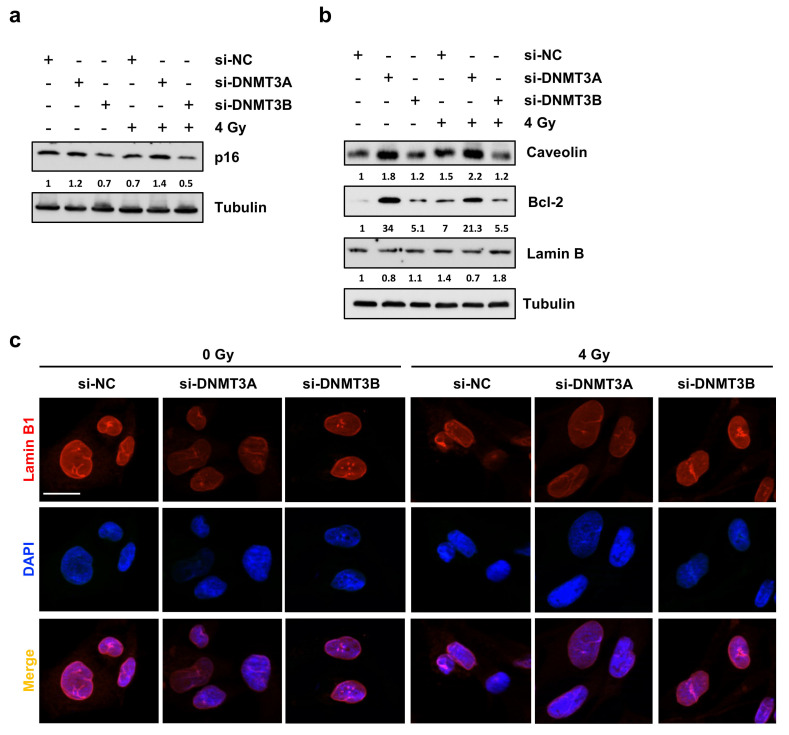
DNMT3A silencing radiosensitizes RD cells by triggering p16-related senescence program. (**a**) Western blotting showing p16 up-regulation in si-DNMT3A RD cells. Tubulin was used as loading control. (**b**) Western blotting assay of specific proteins modulated during the senescence process. Tubulin was used as internal control. (**c**) IF assays showing the expression of Lamin B1 in si-DNMT3A and si-DNMT3B depleted RD cells, also exposed or not to IR. DAPI was used for nuclear staining. Images were acquired by using ApoTome microscope at 40× magnification. Scale bar 20 μm. Figure S1h–i presents the densitometric analyses of the different experiments performed and the corresponding statistical analysis.

Since Giemsa staining highlighted flattened, enlarged, and irregular morphology in si-DNMT3A cells and considering cyclin D1 down-regulation with the concomitant increase of p21 expression levels and the drastic inhibition of colony formation ability, we wondered whether DNMT3A silencing was able to induce senescence in RMS cells. Western blotting assay showed a marked up-regulation of senescence-related marker p16 only in si-DNMT3A sample and the radiation treatment enhanced this activation (Figure 6a).

**Figure 7:**
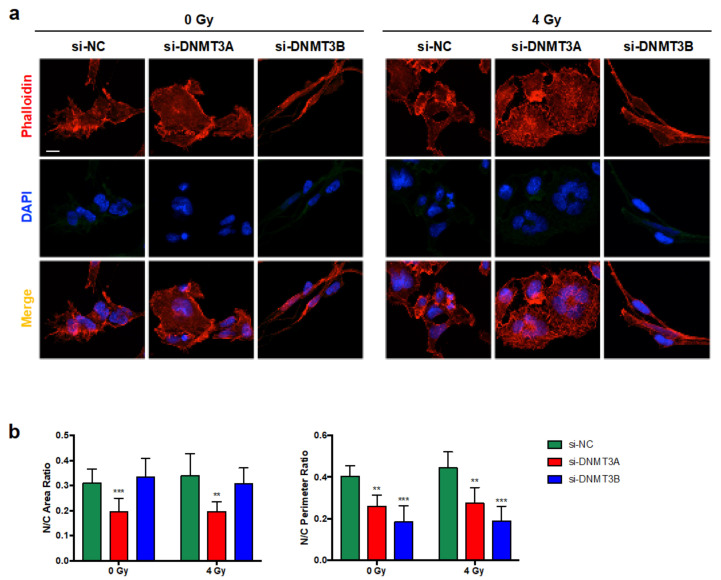
Morphological changes induced by DNMT3A and DNMT3B silencing and radiation treatment in RD cells. (**a**) IF assays showing the expression of Phalloidin in si-DNMT3A and si-DNMT3B depleted RD cells, also exposed or not to IR. DAPI was used for nuclear staining. Images were acquired by using ApoTome microscope at 40× magnification. Scale bar 50 μm. (**b**) Histograms represent the mean value ± SD of nucleus (N)/cytoplasm (C) area (left panel) or perimeter (right panel) ratio calculated by using ImageJ software. Area and perimeter of N and C were measured for each cell in four random merged Phalloidin/DAPI images for the specific silencing (**, *p* < 0.01; ***, *p* < 0.001 vs. si-NC/0 Gy). In multinucleate cells, the nuclear area or perimeter was calculated as the sum of each nucleus.

Thus, we investigated the expression levels of different proteins that can be modulated in senescent cells during the multi-step process, such as Caveolin-1 [31], Lamin B [32], and Bcl-2 [33,34]. As shown in Figure 6b, only DNMT3A depletion with and without IR induced increased expression of Caveolin-1 and Bcl-2 with the simultaneous slight down-regulation of Lamin B, also confirmed by IF experiments (Figure 6c). The same trend of p16 and Bcl-2 modulation was also obtained in JR1 cells, as reported in Figure S2d. Finally, Phalloidin expression analysed by IF assays, in accordance with Giemsa staining, highlighted the deep differences in cellular shape of RD cells transfected with si-DNMT3A compared to si-NC and si-DNMT3B (Figure 7a). Indeed, DNMT3B silencing induced an elongated morphology, whilst after the specific DNMT3A knocking down, RD cells showed a flat and very large shape, peculiar of senescent cells, and the radiation treatment increased these morphological changes (Figure 7a,b).

Altogether, these results indicate that DNMT3A knocking down might synergize with IR by inducing senescence in RMS cells.

### 3.6. Radiation Treatment Preserves the Myogenic Program Triggered by DNMT3B Silencing and Induces Irreversible DNA Damage in Undifferentiated Cells

**Figure 8:**
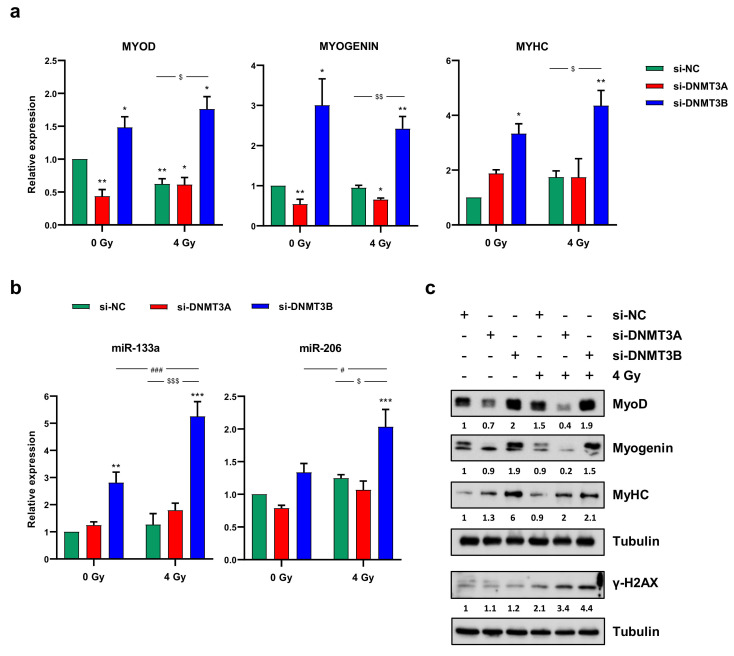
Radiotherapy maintains the myogenic program induced by DNMT3B knocking down. (**a**) Quantitative analysis of differentiation marker MYOD, MYOGENIN and MYHC in RD cells transfected with si-DNMT3A or si-DNMT3B and then exposed to 4 Gy. Data were expressed as fold increase over si-NC/0 Gy, arbitrarily set at 1 and GAPDH was used as endogenous control. Bars represent mean values ± SD of three independent experiments, each performed in triplicate. Statistical analyses were performed by using two-way ANOVA: **, *p* < 0.01; *, *p* < 0.05 vs. si-NC/0 Gy; $$, *p* < 0.01, $, *p* < 0.05 vs. si-NC/4 Gy. (**b**) q-PCR assay of myomiR miR-133a and miR-206 in RD cells at 144 h after transfection with si-DNMT3A or si-DNMT3B and 96 h after radiation treatment. Data were expressed as fold increase over si-NC/0 Gy, arbitrarily set at 1. U6 was used as internal control. Bars represent means ± SD of five independent experiments, each performed in triplicate. Statistical analyses were performed by using two-way ANOVA: ***, *p* < 0.001; **, *p* < 0.01 vs. si-NC/0 Gy; ###, *p* < 0.001, #, *p* < 0.05 vs. si-DNMT3B/0 Gy; $$$, *p* < 0.001, $, *p* < 0.05 vs. si-NC/4 Gy. (**c**) Western blotting assay showing MyoD, Myogenin, MyHC and γ-H2AX up-regulation in RD cells transfected with si-DNMT3B (144 h) and exposed to IR (96 h). Tubulin expression was used as loading control. Figure S1j presents the densitometric analyses of the different experiments performed and the corresponding statistical analysis.

Since in our previously published data we have demonstrated that DNMT3B silencing is able to reactivate the myogenic program in RD cells [25], in this study, we assessed the effects of the radiation therapy on the myogenic differentiation induced by DNMT3B depletion. Primarily, we confirmed that the rescue of the cell phenotype is specifically mediated only by DNMT3B knocking down; indeed, RD cells did not show increased expression of myogenic markers, neither 72 h (data not shown) nor 144 h (Figure 8a–c) after transfection with si-DNMT3A and without irradiation.

**Figure 9:**
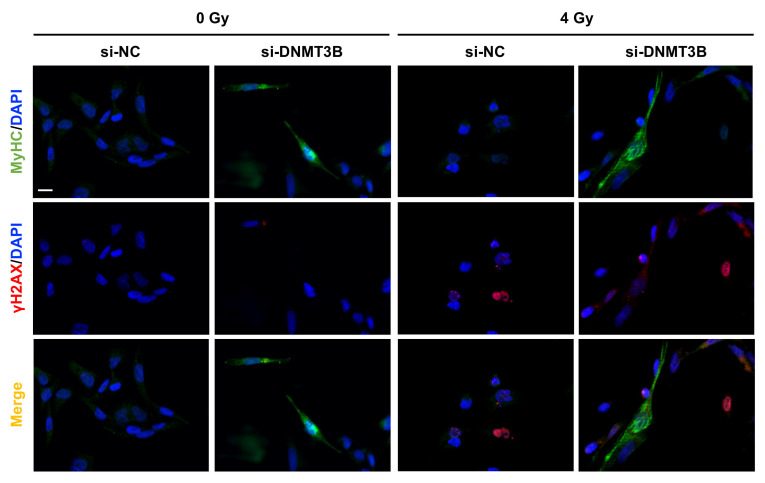
IR induce irreversible DNA damage in undifferentiated si-DNMT3B transfected RD cells. IF assay showing the expression and localization of MyHC and γ-H2AX proteins in RD cells fixed at 7 days after si-DNMT3B transfection and 5 days after irradiation with a single dose of 4 Gy. DAPI was used for nuclear staining. Images were acquired by using ApoTome microscope at 40× magnification. Scale bar 50 μm.

On the contrary, the up-regulation of MyoD, Myogenin and MyHC (Myosin Heavy Chain) expression at both mRNA and protein levels (Figure 8a–c) as well as the increase of two well-established myomiR, miR-133a and miR-206, analysed by q-PCR assay (Figure 8b) detected in si-DNMT3B/4 Gy samples compared to the negative control cells demonstrated that radiation exposure did not affect the myogenic commitment activated by DNMT3B depletion. The reactivation of a proper myogenic differentiation upon DNMT3B knocking down (with and without radiation exposure) was also confirmed in JR1 cells (Figure S2e). Interestingly, si-DNMT3B/4 Gy-treated cells, as showed by Western blotting assay, exhibited a strong γ-H2AX overexpression even 96 h after irradiation (Figure 8c). Thus, we performed immunofluorescence experiments to exclude the co-expression of γ-H2AX and MyHC by the same cells. As displayed by Figure 9, after transfection with si-DNMT3B and radiation treatment, we obtained two different cell population: properly differentiated cells expressing MyHC and not γ-H2AX and damaged cells negative for MyHC and positive for γ-H2AX. All together, these results suggest that the combined treatment DNMT3B silencing/irradiation might be a promising strategy to counteract RMS progression, by inducing irreversible DNA damage mainly in those cells in which it is not reactivated the myogenic program.

## 4. Discussion

RMS is a high-grade, malignant, paediatric solid tumour. Despite the improvement of conventional cures in the last years, the three-year overall survival for children with metastatic and recurrent disease is about 30%. The management of patients with RMS requires a multimodal approach including surgical resection, radiation therapy, and systemic chemotherapy [1]. The molecular mechanisms responsible for therapy resistance are not fully understood; thus, the study of every single molecule involved in therapeutic failure is extremely crucial for the development of more efficacious and less toxic targeted therapies and the identification of novel biomarkers. Recently, we demonstrated the overexpression of DNMT1, DNMT3A, and DNMT3B in RMS tumour biopsies compared to normal skeletal muscle and the oncogenic role, which is played by DNMT3B in RMS [25]. Here, we analysed the effects of DNMT3A silencing in ERMS cells and the ability of the de novo DNMT3A- and DNMT3B-specific depletion to potentiate the efficacy of radiation treatment, since IR alone did not decrease DNMT3A and DNMT3B expression levels. By performing RNAi experiments in RD cells, followed by irradiation with a single dose of 4 Gy, we observed a drastic reduction of cell growth and clonogenic ability in respect to both the single treatments (knocking down alone or IR alone).

However, the two specific gene silencing methods induced different cellular and molecular effects. We found that DNMT3A knocking down enhanced radiotherapy-related effects by triggering senescence process, whilst DNMT3B depletion and IR significantly induced DNA damage by affecting the nucleic acid repair machinery.

Cellular senescence suppresses cancer by driving viable tumour cells in a state of permanent growth arrest without inducing cell death [35]. Stress-induced (premature) senescence can be activated by different stimuli including DNA damage, oxidative stress, chemotherapy, mitochondrial disfunction, and epigenetic changes [32]. After DNMT3A knocking down in RD cells, MTT assay did not show any reduction in cell viability (data not shown), despite the strong inhibition of cell proliferation highlighted by both trypan blue dye exclusion test and clonogenic assay. We hypothesize that DNMT3A restoration triggers cellular senescence stimulating temporary p21-mediated cell cycle arrest followed by p16 activation, which in turn cause permanent cell cycle arrest by promoting the prolonged cyclin/CDK complexes inhibition, as confirmed by the decreased cyclin D1 expression levels. This assumption is also supported by the numerous CpG-rich regions, which are present in the promoter region of the p16 coding gene [36]. Indeed, experimental evidence suggests that DNMT1 and DNMT3B are responsible for p16 promoter hyper-methylation, this leading to its inactivation in several tumours [37,38,39,40]. Our results suggest that in ERMS cells p16-related epigenetic modification could be carried out by DNMT3A, since we did not detect increased p16 expression levels after si-DNMT3B transfection. Caveolin-1 is a structural protein that is required for the formation of caveolae, invaginations of the plasma membrane involved in a variety of cellular processes. In the last years, several studies demonstrated that Caveolin-1 plays a pivotal role in triggering senescence [41]. Dasari et al. showed that oxidative stress-induced senescence might be stimulated by Caveolin-1 transcription, which was promoted by p38 activation [42]. Thus, the increased p-p38 levels, found after DNMT3A silencing with and without irradiation, might be responsible for both p21 and Caveolin-1 overexpression and also for the regulation of the DDR-independent senescence-associated secretory phenotype (SASP), as reported by Freund and colleagues [43]. Finally, the progression from early to full senescence was confirmed by Lamin B down-regulation and elevated Bcl-2 levels. Several studies showed the importance of epigenetic changes in the activation of cellular senescence. A work performed on colorectal cancer cells reported that different concentrations of Doxorubicin are able to induce apoptosis or senescence and that DNMT3A plays a key role in this switch through the regulation of p21 [44]. Moreover, treatment with 5-Aza-2′-deoxycytidine (5-aza), a DNMT inhibitor, was shown to induce senescence in many solid tumours, including osteosarcoma, lung mesothelioma, hepatocellular carcinoma, and breast cancer [40,45,46,47]. Emerging evidence suggests senotherapy (targeted removal of senescent cells, immune-mediated senescent cell clearance, and SASP neutralization) [48,49,50] as a novel anticancer treatment especially in combination with conventional strategies. Indeed, p16 overexpression has been reported to correlate with a better response to radiation therapy in oropharynx cancer [51].

Regarding DNMT3B, we showed that the specific knocking down is able alone to induce a DNA damage in RD cells and this effect, combined with the block of DSB repair machinery, is responsible for the drastic cell growth inhibition observed after the simultaneous treatment with the radiation exposure. Radiosensitivity has been correlated with DSB numbers remaining at 24 h after IR [52] and, in our experiments, we detected the sustained activation of γ-H2AX only in si-DNMT3B/4 Gy samples (Figure 2d), suggesting that irradiated RD cells with elevated DNMT3B levels are able to repair DNA damages. Indeed, 24 h after radiation therapy, si-NC/4 Gy cells showed higher levels of ATM and DNA-PKcs, two of the main DDR signalling kinases [53], and Rad51, directly involved in the DNA repair [54], then si-DNMT3B/4 Gy samples. Generally, formation of γ-H2AX foci maintains the cell cycle checkpoint while DSBs are being repaired [54,55]. If DNA damage cannot be repaired, cells undergo apoptosis [56]. In this study, after DNMT3B silencing and irradiation, we report a drastic G1 cell cycle arrest induced by p21 activation; however, despite the accumulation of γ-H2AX at 72 h after irradiation, we were unable to assess programmed cell death in the long time, likely due to the transient effects of siRNA transfection. However, as previously published by our group, DNMT3B depletion is also able to reactivate the myogenic differentiation and the radiation therapy did not affect this program. Immunofluorescence assays showed γ-H2AX foci only in RD cells that were defective for commitment to terminal differentiation, as confirmed by MyHC negativity. Further experiments will be addressed to better understand the fate of these cells by using stable DNMT3B-silenced RD clones. We speculate that the apoptosis induction failure at 72 h after IR in RD cells not properly differentiated could be due to p53 mutation and inactivation. It could be useful to perform again these experiments in RD cells expressing wild type p53 to understand if the active form of the tumour suppressor protein in combination with DNMT3B depletion and IR is able to trigger apoptosis in RMS cells. Moreover, our data on single siRNA transfections suggest that it may be present a one-sided regulation loop between DNMT3A and DNMT3B proteins, in with DNMT3A being able to directly modulate DNMT3B expression but not the other way. So, double DNMT3A and DNMT3B knocking down needs to be further performed in order to better explore the biological and molecular effects exerted by the concomitant absence of the two enzymes in ERMS cellular context and to set up the best conditions to strongly impact cancer cell proliferation, programmed death and differentiative rescue.

Finally, our studies mainly focus on DNMT3B function in RMS tumours, showing that its silencing is able to induce a sustained activation of p38, this kinase having a key role not only in rescuing myogenesis in RMS cells but also in the modulation of the DNA damage response, mainly in combination with radiation therapy. So, our data open the way to new potential strategies based on DNMT-related functions to improve therapeutic intervention for RMS treatment.

## 5. Conclusions

The present study describes for the first time the potential clinical application of DNMT3A and DNMT3B inhibition as a novel radiosensitizing strategy in RMS cells. Restoration of both DNMT3A and DNMT3B levels might potentiate the effects of IR by respectively inducing senescence or affecting the DNA repair machinery.

## Data Availability

Not applicable.

## Supplementary Materials

The following are available online at https://www.mdpi.com/article/10.3390/cells10112956/s1, Figure S1. Densitometric analysis of the immunoblots performed on RD cells. (**a**–**j**) Densitometric analysis of the immunoblots was performed using Image Lab 5.1 software (Bio-Rad) and bars represent the mean value ± SD of three independent experiments. Data were expressed as fold increase over si-NC/0 Gy, arbitrarily set at 1 and tubulin was used as loading control. Statistical significance: *, *p* < 0.05; **, *p* < 0.01; ***, *p* < 0.001 vs. si-NC/0 Gy; Figure S2. Effects of DNMT3A/3B silencing and radiation treatment on JR1 cells. (**a**) Representative images of JR1 cells stained with Giemsa and photographed at 20× magnification at 144 h after DNMT3A or DNMT3B depletion and 96 h after irradiation. Scale bar 200 μM. (**b**–**e**) Western blotting assays of specific proteins in JR1 cells transfected with si-DNMT3A, si-DNMT3B or si-NC at 24 h after radiation treatment. Tubulin expression was used as loading control. Histograms represent densitometric analysis. Bars are the mean value ± SD of two independent experiments. Data were expressed as fold increase over si-NC/0 Gy, arbitrarily set at 1 and tubulin was used as loading control. Statistical significance: *, *p* < 0.05; **, *p* < 0.01; ***, *p* < 0.001 vs. si-NC/0 Gy; Figure S3. DNMT3A and DNMT3B silencing in combination with IR has no effects on RMS cell migration. Three hours after radiation treatment RD cells, transfected with si-DNMT3A or si-DNMT3B, were plated in serum-free medium in the upper compartment of Cell Culture Inserts with 8 μm pore polycarbonate filters. Representative images of migrated cells stained with crystal violet and photographed at 20× magnification. Cell migration capacity was calculated as fold increase over si-NC/0 Gy, arbitrarily set at 1. Bars represent the mean values ± SD of three independent experiments each performed in triplicate. Statistical analyses were performed by using two-way ANOVA: ***, *p* < 0.001; **, *p* < 0.01 vs. si-NC/0 Gy. Uncropped Western Blot Images.
