# Supplementary figures and images for: DNMT3A and DNMT3B Targeting as an Effective Radiosensitizing Strategy in Embryonal Rhabdomyosarcoma
Source: Cells. 2021 Oct 30;10(11):2956. doi: 10.3390/cells10112956 (PMC8616246; doi:10.3390/cells10112956)

**a**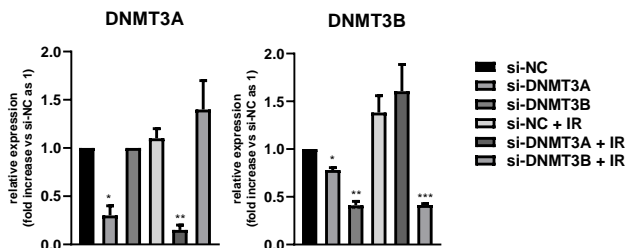**b**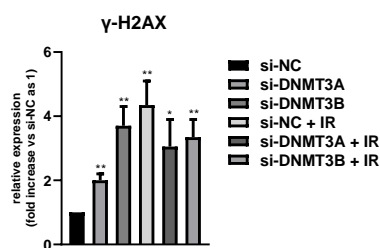**c**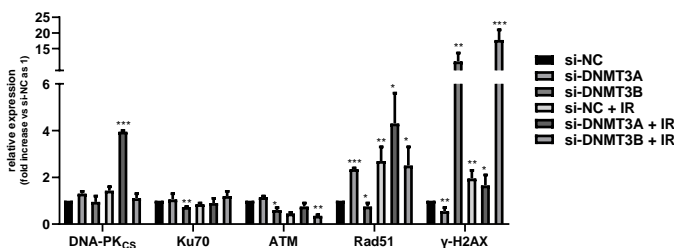**d**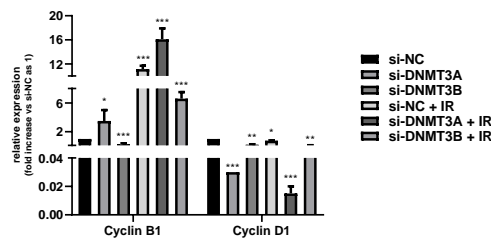**e**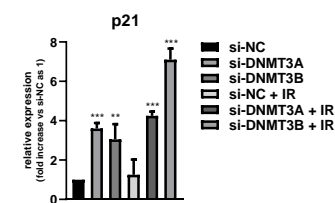**f**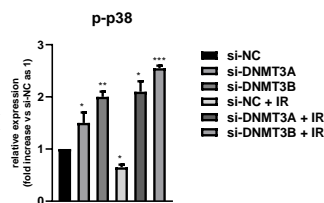**g**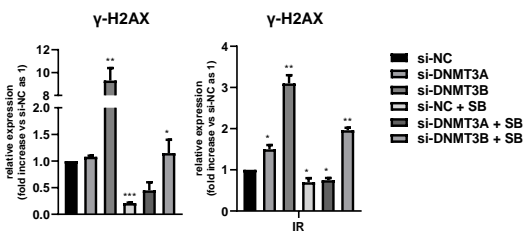**h**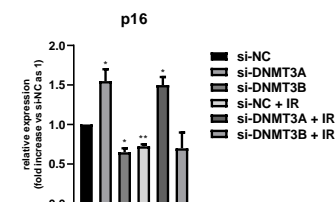**i**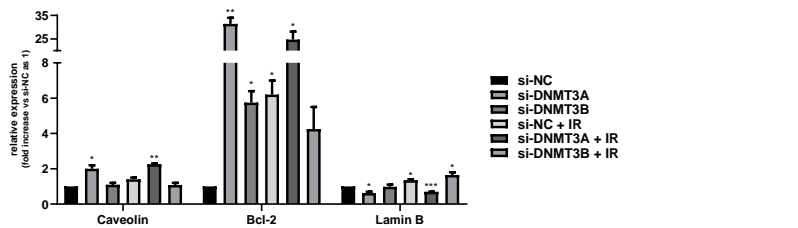**j**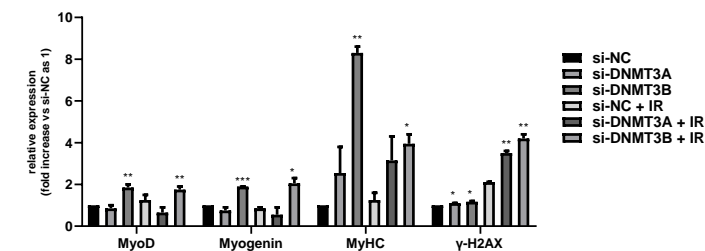

Supplement: Supplementary file 1 [file cells-10-02956-s001.zip › Figure S1_REVISED.pdf]

**a**

si-NC

si-DNMT3A

si-DNMT3B

0 Gy

4 Gy

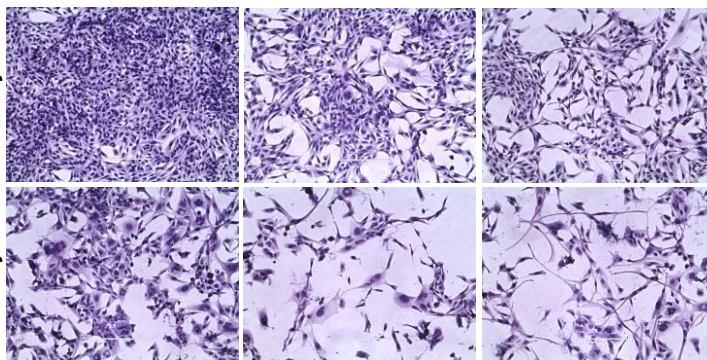**b**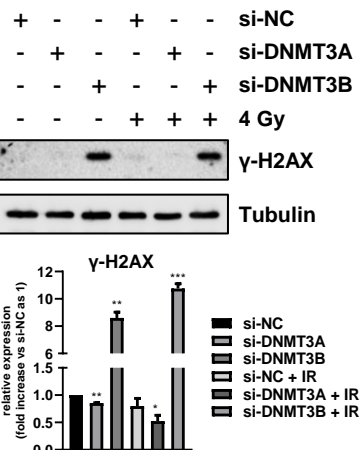**c**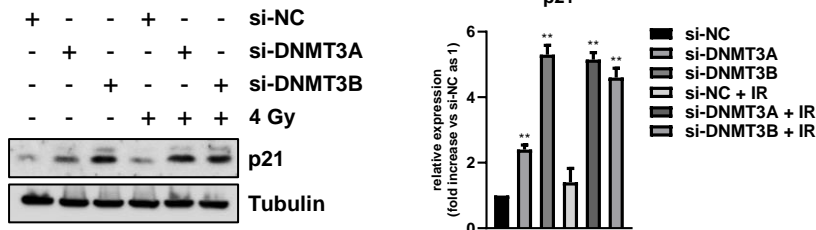**d**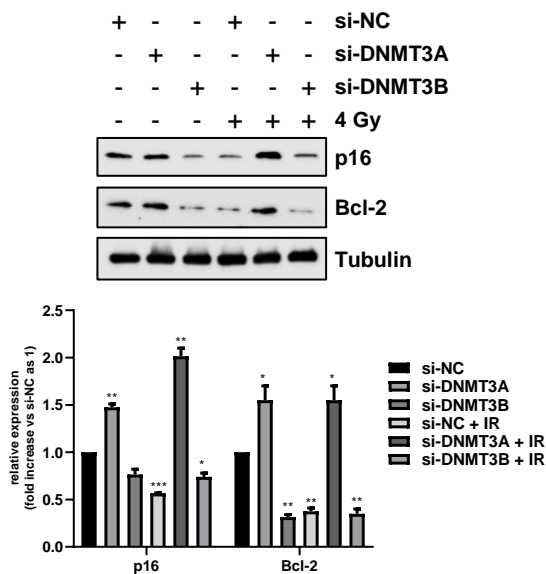**e**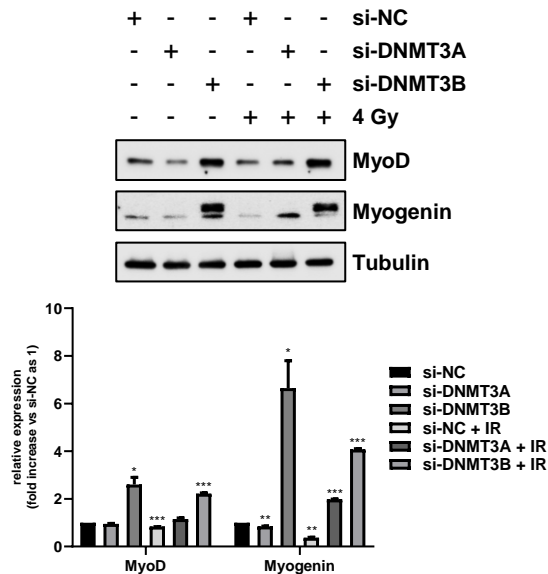

Supplement: Supplementary file 1 [file cells-10-02956-s001.zip › Figure S2_REVISED.pdf]

0 Gy

4 Gy

si-NC

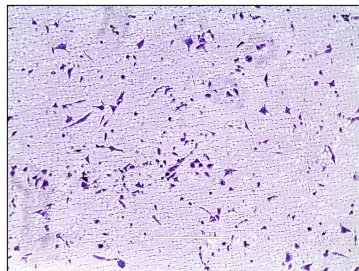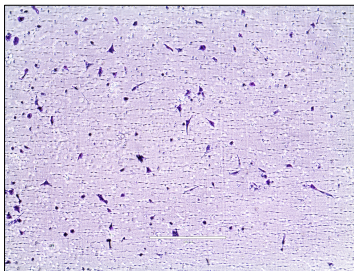

si-DNMT3A

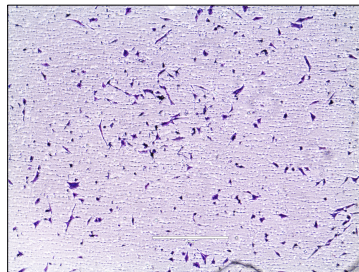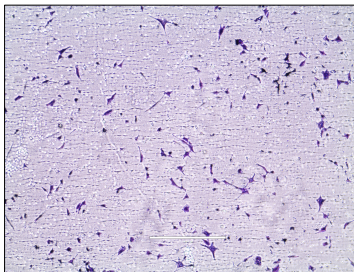

si-DNMT3B

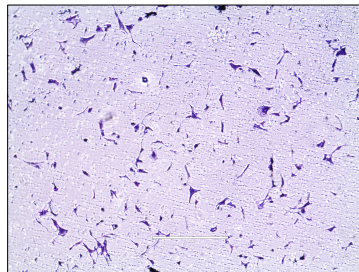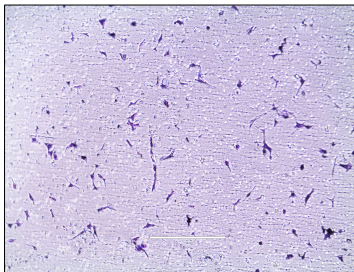

si-NC si-DNMT3A si-DNMT3B

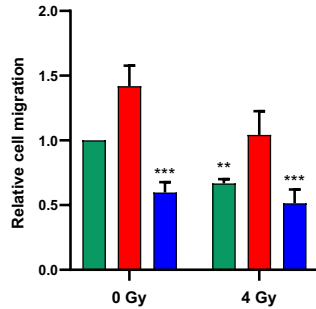

Supplement: Supplementary file 1 [file cells-10-02956-s001.zip › Figure S3_REVISED.pdf]
